# Clinical physiological parameters for the prediction of gram-negative bacterial infection in the emergency department

**DOI:** 10.1186/s12879-020-05758-1

**Published:** 2021-01-13

**Authors:** Chan-Peng Hsu, Hsin-Yu Chen, Wei-Lung Chen, Jiann-Hwa Chen, Chien-Cheng Huang, Po-Han Wu, Jui-Yuan Chung

**Affiliations:** 1 Department of Emergency Medicine, Hsinchu Cathay General Hospital, Hsinchu, Taiwan; 2 Department of Pediatric, Hsinchu Cathay General Hospital, Hsinchu, Taiwan; 3 Department of Emergency Medicine, Chiayi Chang Gung Memorial Hospital, Chiayi, 613 Taiwan; 4 Fu Jen Catholic University School of Medicine, Taipei, Taiwan; 5 Department of Emergency Medicine, Chi-Mei Medical Center, Tainan, Taiwan; 6 Department of Environmental and Occupational Health, College of Medicine, National Cheng Kung University, Tainan, Taiwan; 7 Department of Senior Services, Southern Taiwan University of Science and Technology, Tainan, Taiwan

**Keywords:** Clinical parameters, Gram-negative bacteria infection, Emergency department

## Abstract

**Background:**

Early detection and treatment of Gram-negative bacteria (GNB), major causative pathogens of sepsis (a potentially fatal condition caused by the body’s response to an infection), may benefit a patient’s outcome, since the mortality rate increases by 5–10% for each hour of delayed therapy. Unfortunately, GNB diagnosis is based on bacterial culture, which is time consuming. Therefore, an economic and effective GNB (defined as a positive blood, sputum, or urine culture) infection detection tool in the emergency department (ED) is warranted.

**Methods:**

We conducted a retrospective cohort study in the ED of a university-affiliated medical center between January 01, 2014 and December 31, 2017. The inclusion criteria were as follows: (1) age ≥ 18; (2) clinical suspicion of bacterial infection; (3) bacterial culture from blood, sputum, or urine ordered and obtained in the ED. Descriptive statistics was performed on patient demographic characteristics, vital signs, laboratory data, infection sites, cultured microorganisms, and clinical outcomes. The accuracy of vital signs to predict GNB infection was identified via univariate logistic regression and receiver operating characteristic (ROC) curve analysis.

**Results:**

A total of 797 patients were included in this study; the mean age was 71.8 years and 51.3% were male. The odds ratios of patients with body temperature ≥ 38.5 °C, heart rate ≥ 110 beats per minute, respiratory rate ≥ 20 breaths per minute, and Glasgow coma scale (GCS) < 14, in predicting GNB infection were found to be 2.3, 1.4, 1.9, and 1.6, respectively. The area under the curve values for ROC analysis of these measures were 0.70, 0.68, 0.69, and 0.67, respectively.

**Conclusion:**

The four physiological parameters were rapid and reliable independent predictors for detection of GNB infection.

**Supplementary Information:**

The online version contains supplementary material available at 10.1186/s12879-020-05758-1.

## Background

Gram-negative bacteria (GNB) are more frequently associated than other microbes with severe sepsis and septic shock [1], a potentially life-threatening consequence of the body’s inflammatory response to pathogens. There are two theories regarding how GNB trigger these harmful systemic responses. First, bacteria invade blood vessels and produce inflammatory mediators that circulate throughout the body, resulting in systemic inflammation and multiple organ failure. Second, bacteria and their endotoxins induce local inflammation in extravascular tissues that release immune mediators into the bloodstream [2].

Early recognition of these pathogens and appropriate patient disposition are crucial, as numerous studies have demonstrated benefits to patient outcomes [3–6]. One study indicated that failure to implement effective antibiotic therapy within the first 24 h in GNB sepsis patients may result in a longer hospital stay and a higher mortality rate [7]. To identify sepsis, Severe Inflammatory Response Syndrome (SIRS) criteria were initially established in 1992 by the American College of Chest Physicians (ACCP), and the Society of Critical Care Medicine (SCCM) defined sepsis with four main variables, including body temperature, heart rate, respiratory rate, white blood cell count, and percentage of band form neutrophils [8]. However, the use of SIRS criteria to identify sepsis has fallen out of favor. The predominant tool in current use is the Sequential Organ Failure Assessment (SOFA), which consists of several complex variables, including mean arterial pressure, consciousness level, partial pressure of oxygen, platelet count, creatinine level, and bilirubin level [8]. The quick sepsis-related organ failure assessment (qSOFA) score is the prompt and simple version of the SOFA score, which is able to evaluate the patient at bedside. The qSOFA score includes only three clinical parameters: respiratory rate, altered mental status, and systolic blood pressure [8].

The 2018-sepsis-one-hour bundle suggests early administration of one or more broad-spectrum antibiotics, which are able to treat all likely pathogens of patients with suspected infection; antibiotic stewardship was also emphasized as an essential aspect of high-quality sepsis management [9]. At present, the gold standard to diagnose GNB infection is based on bacterial culture, which may take hours or days before the result is known and can lead to delayed treatment decisions. The inflammatory mediators induced by GNB infection may result in a significant inflammatory response, including fever, tachycardia, and shortness of breath [10]. However, none of the scoring tools, such as qSOFA score and SIRS criteria, had ever been used to predict GNB infection. Given that both of the abovementioned scoring tools involve clinical parameters, we aimed to discover a prompt, simple, and inexpensive bedside prediction tool to detect GNB infection in the emergency department (ED) to assist clinical physicians with timely antibiotic treatment and enable accurate patient disposition.

## Methods

### Study design, setting, and participants

This was a retrospective cohort study, conducted in a university-affiliated medical center (Cathay General Hospital, Taipei) in Northern Taiwan with 40 ED beds and 800 ward beds and approximately 55,000 patients visiting annually. The study period was between January 01, 2014 and December 31, 2017. Patients who fulfilled the following inclusion criteria were included: (1) age above 18 years, (2) suspected bacterial infection, and (3) bacterial culture from blood, sputum or urine, ordered and obtained in the ED. Patients who were transferred from other hospitals, had out-of-hospital cardiac arrest, were pregnant, or had mixed infections were excluded.

### Definition of variables and primary outcome

Suspected bacterial infection is identified by: (1) physician’s clinical judgment through chart review and infection related disease codes, or (2) ED clinical parameters that indicate infection, such as severe inflammatory response syndrome (SIRS) and qSOFA score. SIRS is defined as a heart rate > 90 beats per minute, respiratory rate > 20 breaths per minute, temperature < 36 °C or > 38 °C, white blood cell count < 4000 / mm^3^ or > 12,000 / mm^3^, and band form > 10% [11]. qSOFA score is defined as systolic blood pressure ≤ 100 mmHg, respiratory rate ≥ 22 breaths per minute, and Glasgow Coma Scale < 15 [8]. Positive cultures were defined as: (1) Positive blood culture: at least two bottles of blood culture yielding the same pathogen [12]. Two sets of blood cultures (two aerobic bottles, two anaerobic bottles), were collected from each patient via peripheral venipuncture at two sites, with a 30-min interval between sample collections. (2) Positive sputum culture: pathogen growth in sputum specimens with fewer than 25 squamous epithelial cells per low-power field [13]. Sputum specimen was indicated if a patient had clinical signs, such as productive cough, purulent sputum, dyspnea, or desaturation. Sputum culture was collected by either patient’s expectorated sputum in a sterile container after deep coughs, nasotracheal or orotracheal aspiration, or endotracheal tube aspiration. (3) Positive urine culture: pathogen growth > 10^5^ colony-forming units (CFU) per milliliter in clean-catch midstream urine specimens [14]. Patients with symptoms of dysuria, frequency, urgency, hematuria, suprapubic pain, fever, chills, or flank pain, with or without costovertebral tenderness, were indicated for urine specimen collection. The methods of urine culture collection included self-collection techniques, urethral catheterization, and suprapubic puncture.

Contaminants in cultures were determined as follows: a single positive blood culture with growth of pathogens that represent contamination, including coagulase-negative staphylococci, *Corynebacterium* species, *Bacillus* species, *Propionibacterium acnes*, *Micrococcus* species, viridans group streptococci, enterococci, and *Clostridium perfringens* [12]; urine culture with more than two isolates at greater than or equal to 10 000 CFU/mL [14]; positive sputum cultures in which the isolates consisted of normal oral flora, including *Neisseria catarrhalis*, *Candida albicans*, diphtheroids, alpha-hemolytic streptococci, and some staphylococci [13].

Sepsis is defined as life-threatening organ dysfunction caused by a dysregulated host response to infection, where organ dysfunction can be identified by sepsis-related organ failure assessment (SOFA) score ≥ 2. However, a quick sepsis-related organ failure assessment (qSOFA) score does not require laboratory tests and can be assessed quickly, therefore, in the setting of an emergency department. We used a qSOFA score ≥ 2 to define sepsis [11].

The diagnosis of pneumonia was based on both clinical presentation of productive cough, chest pain, fever, and dyspnea, and pulmonary infiltration visible on the chest image. The indications for chest radiographies were based on both physician’s clinical judgement and patient’s clinical presentation, including productive cough, chest pain, fever, and dyspnea.

### Data collection and assignment to case and control groups

**Fig. 1:**
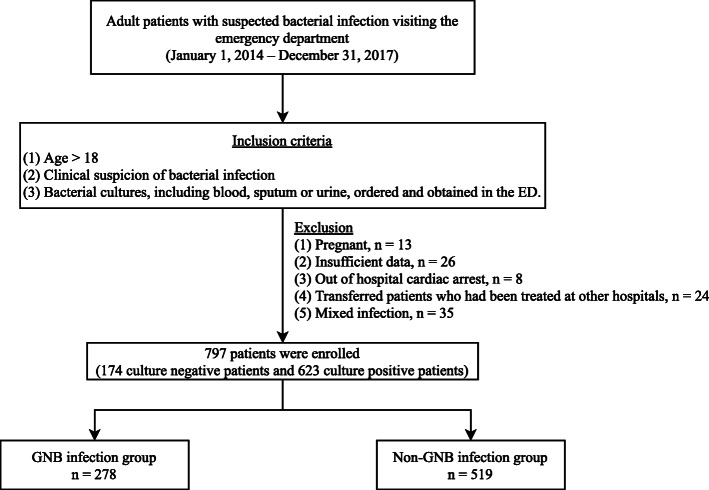
Flowchart of this study

The retrospective chart review method was used to acquire data of patients who fulfilled the inclusion criteria. Demographic characteristics, including vital signs (obtained at the ED triage), laboratory data, infection sites, cultured microorganisms, qSOFA scores, SIRS criteria, and clinical outcomes, were obtained by an emergency physician (Table 1). In total, 903 bacteria-infected ED patients were initially recruited, with a total of 797 patients included (174 culture negative patients and 623 culture positive patients) in the study. Exclusions (106 patients) were made for insufficient data, presence of mixed infections, occurrence of an out-of-hospital cardiac arrest, transferal of patients treated at other hospitals, or pregnant patients (Fig. 1). The recruited patients were further divided into two groups based on the culture result, with 278 patients assigned to the GNB group and 519 patients to the non-GNB group (including 174 culture negative patients). All variables were compared between the two groups, and the accuracy of clinical parameters to predict GNB infection were also analyzed.

**Table 1:** Demographics of emergency department adult patients with suspected bacterial infection

Characteristics	Total patients (*n* = 797)	GNB (*n* = 278)	Non-GNB^a^ (*n* = 519)	*p*-value
Age (mean ± SD)	71.8 ± 17.2	77.0 ± 1.2	69.0 ± 17.5	< 0.01
Male (%)	51.3%	47.5%	53.4%	0.11
Vital signs (mean ± SD)				
Glasgow coma scale	11.5 ± 4.0	10.9 ± 3.8	11.9 ± 4.0	< 0.01
SBP (mmHg)	96.0 ± 37.3	98.0 ± 35.0	94.9 ± 38.5	0.26
Heart rate (n/min)	101.7 ± 28.1	105.0 ± 25.2	100.0 ± 29.5	0.02
Respiratory rate (n/min)	20.8 ± 6.8	21.9 ± 6.6	20.3 ± 6.9	< 0.01
Body temperature (°C)	37.3 ± 3.6	37.7 ± 1.5	37.0 ± 4.3	0.01
Laboratory data (median, IQR)				
WBC (10^3^/mm^3^)	11.6 (8.1–16.6)	12.3 (8.8–17.1)	11.4 (7.9–16.4)	0.13
CRP (mg/dL)	6.6 (2.0–14.8)	7.9 (3.1–17.4)	5.5 (1.3–12.9)	< 0.01
Infection sites (%)				
Pneumonia	26.3%	37.1%	20.6%	< 0.01
Urinary tract infection	22.1%	55.8%	4.0%	< 0.01
Intra-abdominal infection	11.4%	17.3%	8.3%	< 0.01
Soft tissue infection	3.1%	3.2%	3.1%	0.90
Infectious endocarditis	0.8%	1.4%	0.4%	0.10
CNS infection	0.1%	0.4%	0%	0.17
HIV infection	0.4%	0.0%	0.6%	0.20
Bacteremia	14.3%	36.7%	23.3%	< 0.01
Medical history (%)				
Diabetes	13.2%	11.9%	13.9%	0.43
Malignancy	18.1%	16.5%	18.9%	0.41
Chronic kidney disease	12.5%	6.1%	12.0%	< 0.01
Uremia under hemodialysis	2.9%	0.7%	4.9%	< 0.01
COPD	1.6%	2.2%	1.3%	0.39
Liver cirrhosis	4.9%	3.6%	5.6%	0.21
Autoimmune disease	2.0%	2.2%	1.9%	0.82
qSOFA ≥2 (%)	42.7%	56.6%	46.4%	< 0.01
SIRS ≥3 (%)	29.7%	39.6%	24.5%	< 0.01
Mortality (%)	26.7%	27.7%	24.8%	0.37

*GNB* gram-negative bacteria, *SD* standard deviation, *SBP* systolic blood pressure, *IQR* interquartile range, *WBC* white blood cell, *CRP* C-reactive protein, *CNS* central nervous system, *HIV* Human immunodeficiency virus, *COPD* chronic obstructive pulmonary disease, *qSOFA* quick sepsis related organ failure assessment, *SIRS* systemic inflammatory response syndrome

^a^Including culture negative patients

### Ethical statement

This study was approved by the institutional Review Board of the Cathay General Hospital and was conducted according to the Declaration of Helsinki. This was an observational study, and the patients’ data were fully anonymized; the need for informed consent from the patients was waived.

### Statistical analysis

We used SPSS 23.0 for Mac (SPSS Inc., Chicago, IL, USA) to perform statistical analysis. Continuous normally distributed data are presented as mean ± standard deviation (SD), while continuous data that were not normally distributed are presented as median +/− interquartile range (IQR). Categorical variables were presented as percentages. Independent samples *t*-test, the Mann–Whitney, or Wilcoxon test were used to analyze continuous variables. Pearson’s chi-square test or Fisher’s exact test was used for categorical variables. Univariate analysis was performed to evaluate the prediction of GNB infection among the four clinical parameters that showed significant difference (*p* <  0.05) between the GNB and the non-GNB group (Table 2). We calculated the odds ratio of GNB infection in patients with suspected bacterial infection, using 2, 3, or 4 positive clinical parameters. The optimal cut-off point of each clinical parameter used to predict GNB infection was calculated via Youden index. The area under the receiver operating characteristic curve (AUROC) was then used to evaluate GNB prediction discrimination ability (Table 3). Sensitivity, specificity, positive predictive value, and negative predictive value were also calculated to determine the diagnostic accuracy of the four clinical parameters in predicting GNB infection (Table 4).

**Table 2:** Predicting gram-negative bacterial infection by specific clinical physiology parameters, quick sepsis related organ failure assessment score and systemic inflammatory response syndrome criteria of adult patients with suspected bacterial infection in emergency department, identified by logistic regression

	Odds ratio	95% CI	*p*-value
BT ≥ 38.5 °C	2.30	1.90–3.83	< 0.01
RR ≥ 20/min	1.91	1.51–2.92	< 0.01
GCS < 14	1.57	1.38–2.50	< 0.01
HR ≥ 110 beats/min	1.38	1.27–2.30	0.04
qSOFA ≥2	1.47	1.07–1.97	0.01
SIRS ≥3	1.81	1.33–2.55	< 0.01

*BT* body temperature, *RR* respiratory rate, *GCS* Glasgow coma scale, *HR* heart rate

**Table 3:** Adjusted AUROC for gram-negative bacterial infection prediction by clinical physiology parameters of adult patients with suspected bacterial infection in emergency department

	AUROC	95% CI	*p*-value
BT ≥ 38.5 °C	0.70	0.66–0.74	< 0.01
RR ≥ 20 /min	0.69	0.65–0.72	< 0.01
GCS < 14	0.68	0.64–0.72	< 0.01
HR ≥ 110 beats/min	0.67	0.63–0.71	< 0.01

*AUROC* Area under the Receiver Operating Characteristic, *BT* body temperature, *RR* respiratory rate, *GCS* Glasgow coma scale, *HR* heart rate

**Table 4:** Performance of the clinical physiology parameters in predicting gram-negative bacterial infection in adult patients with suspected bacterial infection in emergency department

	Sensitivity	Specificity	Positive predictive value	Negative predictive value
BT ≥ 38.5 °C	0.32 (0.26–0.37)	0.85 (0.82–0.88)	0.54 (0.46–0.61)	0.70 (0.66–0.74)
RR ≥ 20 breaths/min	0.50 (0.44–0.56)	0.67 (0.63–0.71)	0.45 (0.39–0.50)	0.71 (0.67–0.75)
GCS < 14	0.60 (0.54–0.66)	0.55 (0.51–0.59)	0.42 (0.37–0.47)	0.72 (0.68–0.77)
HR ≥ 110 beats/min	0.45 (0.39–0.51)	0.67 (0.64–0.72)	0.43 (0.37–0.48)	0.70 (0.66–0.74)

*BT* body temperature, *RR* respiratory rate, *GCS* Glasgow coma scale, *HR* heart rate

## Results

A total of 797 patients were included in this study. The male to female ratio was approximately equal. The mean ± SD age was 71.8 ± 17.2 years, and the GNB group was older than the non-GNB group at 77.0 ± 1.2 and 69.0 ± 17.5 years, respectively. Glasgow coma scale (GCS), heart rate, respiratory rate, and body temperature were significantly different (*p*-value < 0.01, 0.02, < 0.01, 0.01) between GNB and non-GNB group. The mean ± SD of GCS was lower in the GNB group (10.9 ± 3.8) than the non-GNB group (11.9 ± 4.0). Heart rate, respiratory rate, and body temperature were higher in the GNB group than the non-GNB group for 105.0 ± 25.2, 21.9 ± 6.6, 37.7 ± 1.5, and 100.0 ± 29.5, 20.3 ± 6.9, 37.0 ± 4.3, respectively.

Meanwhile, significant elevation of heart rate, respiratory rate and body temperature were noted in the culture positive patients (102.3 ± 27.2, 22.0 ± 5.8, 37.3 ± 4.5) than the culture negative patients (97.8 ± 34.0, 20.1 ± 9.9, 36.4 ± 3.1); GCS was 11.2 ± 4.3 in the culture positive patients, which was significantly lower than the culture negative patients. Lower systolic blood pressure was also noted in the culture positive patients than the culture negative patients for 91.4 ± 42.1 and 96.0 ± 37.2 (Supplementary Table 1).

**Fig. 2:**
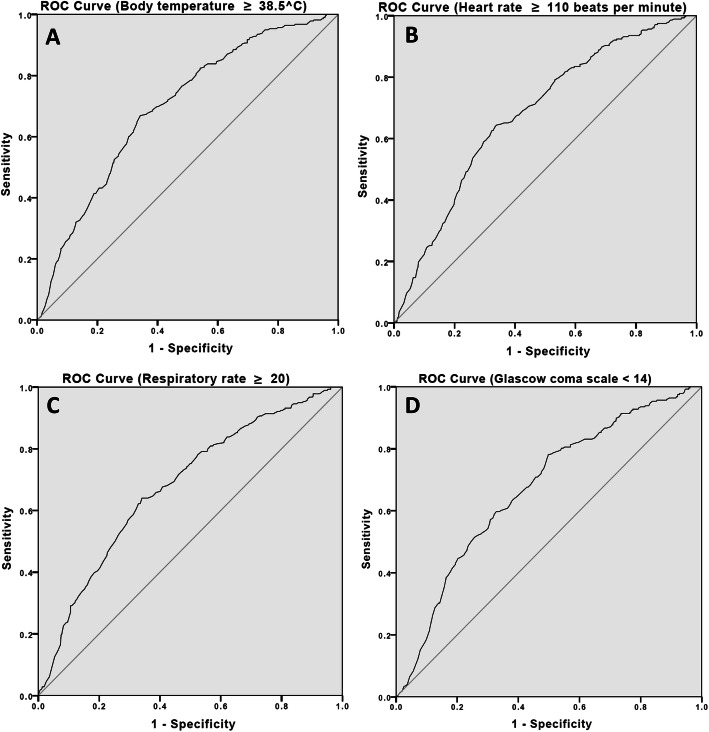
**a**. Area under the curve of body temperature ≥ 38.5 °C; **b**. heart rate ≥ 110 beats per minute; **c**. respiratory rate ≥ 20 breaths per minute; **d**. Glasgow coma scale < 14 to predict gram-negative bacterial infection in the emergency department

The median C-reactive protein (CRP) was 7.9 (IQR: 3.1–17.4) in the GNB group, which was higher than that in the non-GNB group, 5.5 (IQR: 1.3–12.9). The prevalence of pneumonia, urinary tract infection, intra-abdominal infection, and bacteremia was moderately higher in the GNB group than in the non-GNB group at 37.1, 55.8, 17.3, and 36.7%, respectively. The percentage of qSOFA score ≥ 2 and SIRS criteria ≥3 were both significantly higher in the GNB group, at 50.7 and 39.6%, respectively, than the non-GNB group, at 38.3 and 24.5%, respectively. The most frequently identified bacterium from the blood and the urine specimen was *Escherichia coli* (38%); while the most frequently identified pathogen from the sputum was *Pseudomonas aeruginosa* (40%) (Fig. 2).

Comparing bacteremic patients with non-bacteremic patients, regardless of GNB status, bacteremic patients had a significantly higher heart rate, respiratory rate, and body temperature than non-bacteremic patients at 105.3 ± 24.2, 21.6 ± 6.5, and 37.8 ± 1.4, respectively. The mortality percentage in bacteremic patients, regardless of GNB status, was 24.0%; it was 28.2% for the non-bacteremic patients, *p*-value = 0.22. Bacteremic patients had significantly higher percentages for quick sepsis-related organ failure assessment (qSOFA) score ≥ 2 and severe inflammatory response syndrome (SIRS) ≥ 3 at 47.3 and 38.5%, respectively, than the non-bacteremic patients at 40.1 and 25.1%, respectively (Supplementary Table 2). Results showed a higher rate of pneumonia, urinary tract infection, soft tissue infection, and bacteremia in septic patients than in non-septic patients. Furthermore, mortality was significantly higher in the septic patients at 55.4%, than in the non-septic patients at 38.0% (Supplementary Table 3).

**Fig. 3:**
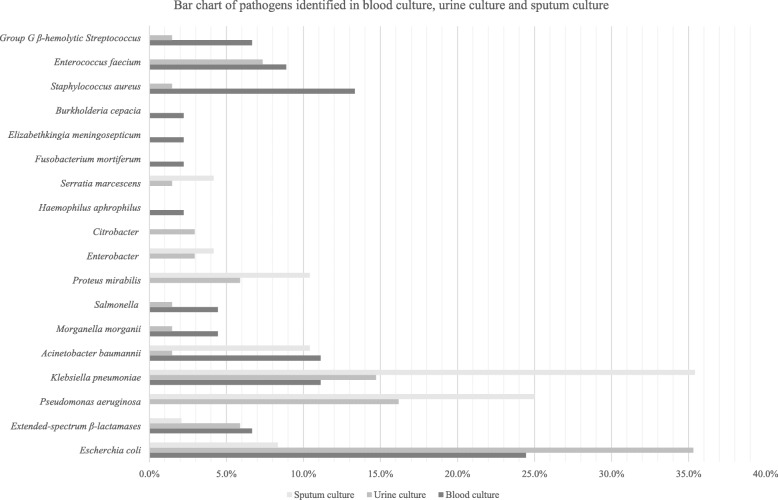
Bar chart of pathogens identified in blood culture, urine culture, and sputum culture

The mortality percentage was higher in the GNB group (27.7%) than the non-GNB group (24.8%). The optimal cut-off point for the clinical parameters to predict GNB infection among ED adult bacteria-infected patients, calculated via Youden index, are as follows: body temperature ≥ 38.5 °C, heart rate ≥ 110 beats per minute, respiratory rate ≥ 20 breaths per minute, and GCS < 14. Logistic regression showed that patients with each of these parameters had, respectively, a 2.3-, 1.4-, 1.9-, and 1.6-fold greater risk of GNB infection than the patients who did not meet the cut-off point of the four study clinical parameters (multivariate analyses showed that patients with body temperature ≥ 38.5 °C, heart rate ≥ 110 beats per minute, respiratory rate ≥ 20 breaths per minute, and GCS < 14 had a 2.44-,1.36-, 1.92-, and 0.73-fold greater risk of GNB infection than the patients who did not meet the cut-off point of the four study clinical parameters [Supplementary Table 4]). Meanwhile, qSOFA ≥2 and SIRS ≥3 had a 1.47- and 1.81-fold greater risk of GNB infection, respectively (Table 2). Diagnostic accuracy tests of the four clinical parameters in predicting GNB infection showed GCS < 14 with the highest sensitivity and negative predictive values of 0.6 and 0.72, respectively; while BT ≥ 38.5 °C had the highest specificity and positive predictive values of 0.85 and 0.54, respectively. All four clinical parameters had approximately equal negative predictive values of 0.70–0.72 (Table 4). The AUROC, adjusted by age (*p* <  0.01), chronic kidney disease (CKD) (*p* <  0.01) and uremia under dialysis, in predicting GNB infection among ED adult bacteria-infected patients, showed body temperature ≥ 38.5 °C and had an acceptable discrimination ability at 0.7 (0.66–0.74) (Fig. 3). We calculated the odds ratio of GNB infection in suspected bacterial infection patients with 2, 3, or 4 of the study clinical parameters showing positive results. Results showed an increasing trend of the odds ratio as the numbers of the study clinical parameters that were positive increased. The odds ratios of GNB infection were 1.79, 2.29, and 4.32 in patients with 2, 3, or 4 of the study clinical parameters showing positive, respectively (Supplementary Table 5). While the odds ratio of GNB combined with sepsis in patients with 2, 3, or 4 of the study indicators showing positive results were 4.02, 4.83, and 5.50, respectively (Supplementary Table 6).

## Discussion

It is crucial to quickly identify the pathogens in septic patients and make a prompt decision in a busy ED, as the mortality rate may increase up to 10% with every hour of delayed diagnosis [15]. Unlike the time-consuming blood culture, assessment of clinical physiological parameters has the advantage of being immediate and easy to acquire in the ED. SIRS criteria and qSOFA scores were both used to identify sepsis via clinical parameters. However, the relative accuracy of these tools for the identification of sepsis remains under debate [16], and none of these have been used to predict GNB infection yet.

This study identified the key clinical physiological parameters that could predict GNB infection in the ED by reviewing medical records of adult patients with suspected bacterial infection, according to the definition mentioned in the *Methods*. Although two of the qSOFA score variables, GCS and respiratory rate, and three of the SIRS criteria variables, heart rate, body temperature, and respiratory rate, were correlated to GNB infection, the cut-off points were different from the qSOFA score and SIRS criteria, as the clinical parameters that predicted GNB infection were: body temperature 38.5 °C, heart rate ≥ 110 beats per minute, respiratory rate ≥ 20 breaths per minute, and GCS < 14. These further indicate that GNB infection may stimulate both severe inflammatory response (body temperature ≥ 38.5 °C, heart rate ≥ 110 beats per minute, respiratory rate ≥ 20 breaths per minute) and cause organ dysfunction (GCS < 14). We determined that the cut-off points of body temperature of ≥38.5 °C and heart rate ≥ 110 beats per minute were higher than the SIRS criteria of > 38 °C and > 90 beats per minute. These were probably related to the systemic inflammation, which could include effects induced by the GNB, such as increased vascular permeability, leukocyte-endothelial adhesion, and neuroendocrine dysregulation [2]. The circulating GNB endotoxin may further precipitate organ dysfunction and shock status, with consequences of poor perfusion to the central nervous system and changes in GCS [2]. In this study and other reports, the respiratory tract was one of the most common infection sites for GNB [17], resulting in pneumonia and increased respiratory rates. Despite the fact that GNB infection may be more likely to develop into sepsis, 43.3% of the GNB infected patients did not fulfill the criteria of qSOFA ≥2. Furthermore, the odds ratio of qSOFA ≥2 in predicting GNB infection was 1.47, which was lower than the individual clinical parameters of body temperature ≥ 38.5 °C, respiratory rate ≥ 20 breaths per minute, and GCS < 14, at 2.30, 1.91, and 1.57, respectively. Although both GNB and non-GNB groups included substantial numbers of patients with elevated sepsis scores and fatal outcomes, the GNB group has statistically significantly greater disease severity than the non-GNB group, as higher percentage of qSOFA ≥2 and SIRS ≥3 were noted in the GNB group.

Prior medical history did not show a significant difference between the GNB and the non-GNB group, making it difficult to predict GNB infection using these methods. However, higher prevalence of CKD and uremia under dialysis were noted in the non-GNB group. A similar finding was noted in a previous study conducted by Berman and colleagues, who concluded that instead of GNB, the major pathogens in infected dialysis patients were gram-positive cocci [18]. In patients with CKD, *Escherichia coli* and *Staphylococcus aureus* accounted for the majority of bloodstream infections, while *Staphylococcus aureus* was especially prominent in CKD patents with an estimated glomerular filtration rate < 30 mL per minute per 1.73 m^2^ [19].

This study demonstrated a significantly higher CRP level in the GNB group than the non-GNB group. Ryuzo and colleagues discovered a similar result, where CRP and interleukin-6 were higher in gram-negative bacteremia than in gram-positive bacteremia in intensive care unit patients [1]. Another study also showed CRP and procalcitonin as effective predictors of bloodstream GNB infection [20]. Although laboratory data may act as potential indicators of GNB infection, it still requires time to obtain the result, and this may jeopardize the early treatment strategy. Furthermore, despite several newly developed molecular rapid diagnostic tools being available for the early detection of these pathogen species, these tools are not widely used in most medical facilities due to their unavailability and expense [21–24].

We discovered that GCS < 14 had the highest sensitivity at 0.6, and BT ≥ 38.5 °C had the highest specificity at 0.85. The negative predictive values of the four study clinical parameters were 0.70–0.72, which indicated that patients who did not meet the cut-off point of the four study clinical parameters were less likely to be infected with GNB. Furthermore, the risk of GNB infection may increase by 4-fold, while when 2, 3, or 4 of the study clinical parameters showed positive, the odds ratios were 1.79, 2.29, and 4.32, respectively. The need to treat for sepsis as well as GNB infection should be considered in patients with 2, 3, or 4 of the study clinical parameters showing positive results, as the odds ratios were 4.02, 4.83, and 5.50, respectively.

To our knowledge, this was the first study conducted to evaluate physiological parameters to predict GNB infection in the ED. However, this study also has some limitations. First, some valuable information and data may be missing due to the nature of a retrospective study setting. Furthermore, due to the retrospective setting of this study, much greater heterogeneity of clinical and laboratory findings was noted in the non-GNB group than the GNB group. It is also difficult to ensure external validity for the larger population of GNB infection, that further studies are needed to validate the results of this study. Second, this was a single-center study and thus the bacterial species detected may have a selection bias. Third, the study was conducted in a tertiary medical center, where the disease severity of the visiting patients may be higher. Fourth, several unmeasured confounding factors may exist, such as the severity of underlying diseases and the time of treatment. Fifth, clinical parameters were obtained only once after arrival at the ED, while initial vital signs may better reflect the original patient status rather than the vital signs obtained after treatment. Sixth, false negative cases may be left out; however, the false negative rate was very low, as Peretz and colleagues discovered that only 0.13% of the initially negative blood cultures were positive after further examination by Gram staining [25]. Although the odds ratio for any combination of the two or three clinical parameters to predict GNB infection were provided in the Supplementary Table 5, we did not calculate the odds ratio for each possible combinations of the two-parameter or three parameter model (there are six possible two-parameter combinations and four possible three-parameter combinations), therefore, developing a clinical physiology scoring tool to predict GNB infection based on the findings of this research and comparing the newly established tool with the existing scoring systems, such as qSOFA and SIRS criteria, will be our future goal.

## Conclusion

Clinical physiological parameters, including body temperature ≥ 38.5 °C, heart rate ≥ 110 beats per minute, respiratory rate ≥ 20 breaths per minute, and GCS < 14, were four rapid, simple, and cost-effective independent predictors that could detect GNB infection early in the ED.

## Supplementary Information

**Additional file 1: Table S1.** Demographics of culture positive and culture negative patients among those with suspected bacterial infection in the emergency department. **Table S2.** Demographics of bacteremic and non-bacteremic patients among those with suspected bacterial infection in the emergency department. **Table S3.** Demographics of septic and non-septic patients among those with suspected bacterial infection in the emergency department. **Table S4.** Multivariate analysis of specific clinical physiology parameters predicting gram-negative bacterial infection in adult patients with suspected bacterial infection in the emergency department. **Table S5.** Logistic regression analysis identifying the number of positive clinical physiology parameters predicting gram-negative bacterial infection in adult patients with suspected bacterial infection in the emergency department. **Table S6.** Logistic regression analysis identifying the number of positive clinical physiology parameters predicting gram-negative bacterial infection with sepsis in adult patients with suspected bacterial infection in the emergency department.

## Data Availability

All data generated or analyzed during this study were included in this manuscript.

## Abbreviations

GNB: Gram-negative bacteria
SIRS: Severe Inflammatory Response Syndrome
ACCP: American College of Chest Physicians
SCCM: Society of Critical Care Medicine
SOFA: Sequential Organ Failure Assessment
qSOFA: Quick sepsis-related organ failure assessment
ED: Emergency department
CFU: Colony-forming unit
SD: Standard deviation
IQR: Interquartile range
AUROC: Area under the receiver operating characteristic curve
GCS: Glasgow coma scale
CRP: C-reactive protein
CKD: Chronic kidney disease
