# Supplementary material for: Clinical physiological parameters for the prediction of gram-negative bacterial infection in the emergency department
Source: BMC Infect Dis. 2021 Jan 13;21:66. doi: 10.1186/s12879-020-05758-1 (PMC7805130; doi:10.1186/s12879-020-05758-1)
Supplement: Supplementary file 1 — Additional file 1: Table S1. Demographics of culture positive and culture negative patients among those with suspected bacterial infection in the emergency department. Table S2. Demographics of bacteremic and non-bacteremic patients among those with suspected bacterial infection in the emergency department. Table S3. Demographics of septic and non-septic patients among those with suspected bacterial infection in the emergency department. Table S4. Multivariate analysis of specific clinical physiology parameters predicting gram-negative bacterial infection in adult patients with suspected bacterial infection in the emergency department. Table S5. Logistic regression analysis identifying the number of positive clinical physiology parameters predicting gram-negative bacterial infection in adult patients with suspected bacterial infection in the emergency department. Table S6. Logistic regression analysis identifying the number of positive clinical physiology parameters predicting gram-negative bacterial infection with sepsis in adult patients with suspected bacterial infection in the emergency department. [file 12879_2020_5758_MOESM1_ESM.docx]

**Table 1. Demographics of culture positive and culture negative patients among those with suspected bacterial infection in the emergency department.**

| Characteristics | Culture positive patients  (n = 623) | Culture negative patients  (n = 174) | *p*-value |
| --- | --- | --- | --- |
| Age (mean ± SD) | 72.3 ± 16.9 | 70.5 ± 17.9 | 0.13 |
| Male (%) | 48.5% | 61.5% | 0.03 |
| Vital signs (mean ± SD) |  |  |  |
| Glasgow coma scale | 11.2 ± 4.3 | 13.4 ± 3.9 | 0.04 |
| SBP (mmHg) | 91.4 ± 42.1 | 96.0 ± 37.2 | 0.16 |
| Heart rate (n/min) | 102.3 ± 27.2 | 97.8 ± 34.0 | 0.02 |
| Respiratory rate (n/min) | 22.0 ± 5.8 | 20.1 ± 9.9 | <0.01 |
| Body temperature (°C) | 37.3 ± 4.5 | 36.4 ± 3.1 | 0.01 |
| Medical history (%) |  |  |  |
| Diabetes | 13.2% | 13.2% | 0.98 |
| Malignancy | 16.7% | 23.0% | 0.06 |
| Chronic kidney disease | 11.7% | 15.5% | 0.18 |
| Uremia under hemodialysis | 2.4% | 4.6% | 0.13 |
| COPD | 1.9% | 0.6% | 0.21 |
| Liver cirrhosis | 4.3% | 6.9% | 0.16 |
| Autoimmune disease | 2.6% | 0.0% | 0.03 |
| qSOFA ≥ 2 (%) | 52.0% | 37.9% | 0.01 |
| SIRS ≥ 3 (%) | 43.1% | 27.3% | <0.01 |
| Mortality (%) | 30.1% | 14.3% | <0.01 |

SD, standard deviation; SBP, systolic blood pressure; COPD, chronic obstructive pulmonary disease; qSOFA, quick sepsis-related organ failure assessment; SIRS, systemic inflammatory response syndrome.

**Table 2. Demographics of bacteremic and non-bacteremic patients among those with suspected bacterial infection in the emergency department.**

| Characteristics | Bacteremic patients  (n = 283) | Non-bacteremic patients  (n = 514) | *p*-value |
| --- | --- | --- | --- |
| Age (mean ± SD) | 75.9 ± 15.4 | 69.5 ± 17.7 | 0.02 |
| Male (%) | 46.3% | 54.1% | 0.04 |
| Vital signs (mean ± SD) |  |  |  |
| Glasgow coma scale | 11.3 ± 3.7 | 11.7 ± 4.2 | 0.29 |
| SBP (mmHg) | 98.5 ± 35.2 | 94.6 ± 38.4 | 0.16 |
| Heart rate (n/min) | 105.3 ± 24.2 | 99.8 ± 30.0 | 0.01 |
| Respiratory rate (n/min) | 21.6 ± 6.5 | 20.4 ± 7.0 | 0.01 |
| Body temperature (°C) | 37.8 ± 1.4 | 37.1 ± 4.3 | 0.01 |
| Medical history (%) |  |  |  |
| Diabetes | 9.5% | 15.2% | 0.02 |
| Malignancy | 15.9% | 19.3% | 0.24 |
| Chronic kidney disease | 10.2% | 13.8% | 0.15 |
| Uremia under hemodialysis | 2.1% | 3.3% | 0.34 |
| COPD | 1.8% | 1.6% | 0.82 |
| Liver cirrhosis | 3.2% | 5.8% | 0.09 |
| Autoimmune disease | 2.5% | 1.8% | 0.49 |
| qSOFA ≥ 2 (%) | 47.3% | 40.1% | 0.05 |
| SIRS ≥ 3 (%) | 38.5% | 25.1% | <0.01 |
| Mortality (%) | 24.0% | 28.2% | 0.20 |

SD, standard deviation; SBP, systolic blood pressure; COPD, chronic obstructive pulmonary disease; qSOFA, quick sepsis-related organ failure assessment; SIRS, systemic inflammatory response syndrome.

**Table 3. Demographics of septic and non-septic patients among those with suspected bacterial infection in the emergency department.**

| Characteristics | Septic  patients  (n = 340) | Non-septic patients  (n = 457) | *p*-value |
| --- | --- | --- | --- |
| Age (mean ± SD) | 77.8 ± 14.9 | 67.3 ± 17.4 | <0.01 |
| Male (%) | 46.3% | 54.1% | 0.04 |
| Vital signs (mean ± SD) |  |  |  |
| Glasgow coma scale | 9.2 ± 3.8 | 13.3 ± 3.2 | <0.01 |
| SBP (mmHg) | 86.3 ± 33.3 | 103.2 ± 27.2 | <0.01 |
| Heart rate (n/min) | 101.4 ± 32.9 | 102 ± 24.1 | 0.76 |
| Respiratory rate (n/min) | 22.9 ± 9.7 | 19.3 ± 2.4 | <0.01 |
| Body temperature (°C) | 37.5 ± 5.3 | 37.1 ± 1.4 | 0.21 |
| Infection sites (%) |  |  |  |
| Pneumonia | 37.9% | 17.7% | <0.01 |
| Urinary tract infection | 25.3% | 19.7% | 0.05 |
| Intra-abdominal infection | 6.8% | 14.9% | <0.01 |
| Soft tissue infection | 3.2% | 3.1% | 0.89 |
| Infectious endocarditis | 0.6% | 0.9% | 0.64 |
| CNS infection | 0.3% | 0% | 0.25 |
| HIV infection | 0.3% | 0.4% | 0.70 |
| Bacteremia | 39.4% | 32.6% | 0.05 |
| Medical history (%) |  |  |  |
| Diabetes | 11.5% | 14.4% | 0.22 |
| Malignancy | 17.6% | 18.4% | 0.79 |
| Chronic kidney disease | 11.2% | 13.6% | 0.31 |
| Uremia under hemodialysis | 1.8% | 3.7% | 0.10 |
| COPD | 2.6% | 0.9% | 0.05 |
| Liver cirrhosis | 2.4% | 6.8% | <0.01 |
| Autoimmune disease | 0.9% | 2.8% | 0.05 |
| Mortality (%) | 55.4% | 38.0% | <0.01 |

SD, standard deviation; SBP, systolic blood pressure; CNS, central nervous system; HIV, Human immunodeficiency virus; COPD, chronic obstructive pulmonary disease

**Table 4. Multivariate analysis of specific clinical physiology parameters predicting gram-negative bacterial infection in adult patients with suspected bacterial infection in the emergency department.**

| Clinical physiology parameters | Odds ratio | 95% CI | *p*-value |
| --- | --- | --- | --- |
| BT ≥ 38.5 °C | 2.44 | 1.70–3.51 | < 0.01 |
| RR ≥ 20/min | 1.92 | 1.39–2.56 | < 0.01 |
| GCS < 14 | 0.73 | 0.54–0.99 | 0.04 |
| HR ≥ 110 beats/min | 1.36 | 0.99–1.87 | 0.04 |

BT, body temperature; RR, respiratory rate; GCS, Glasgow coma scale; HR, heart rate; CI, confidence interval

**Table 5. Logistic regression analysis identifying the number of positive clinical physiology parameters predicting gram-negative bacterial infection in adult patients with suspected bacterial infection in the emergency department.**

| Number of positive clinical physiology parameters | Odds ratio | 95% CI | *p*-value |
| --- | --- | --- | --- |
| 2 | 1.79 | 1.10–2.91 | < 0.01 |
| 3 | 2.29 | 1.32–3.97 | 0.04 |
| 4 | 4.32 | 1.72–10.86 | 0.04 |

CI, confidence interval

**Table 6. Logistic regression analysis identifying the number of positive clinical physiology parameters predicting gram-negative bacterial infection with sepsis in adult patients with suspected bacterial infection in the emergency department.**

| Number of positive clinical physiology parameters | Odds ratio | 95% CI | *p*-value |
| --- | --- | --- | --- |
| 2 | 4.02 | 1.83–8.79 | < 0.01 |
| 3 | 4.83 | 2.11–11.09 | < 0.01 |
| 4 | 5.50 | 1.76–17.15 | 0.03 |

CI, confidence interval
